# Amivantamab治疗*EGFR*/*MET*基因异常非小细胞肺癌的单中心经验

**DOI:** 10.3779/j.issn.1009-3419.2022.102.26

**Published:** 2022-07-20

**Authors:** 静静 王, 雨佳 迟, 含笑 陈, 博 郏, 晓宇 翟, 梦磊 马, 俭杰 李, 明磊 卓

**Affiliations:** 100142 北京，北京大学肿瘤医院暨北京市肿瘤防治研究所胸部肿瘤内一科，恶性肿瘤发病机制及转化研究教育部重点实验室 Key laboratory of Carcinogenesis and Translational Research (Ministry of Education/Beijing), Department of Thoracic Medical Oncology, Peking University Cancer Hospital & Institute, Beijing 100142, China

**Keywords:** 肺肿瘤, EGFR, MET, 靶向治疗, Amivantamab, Lung neoplasms, EGFR, MET, Target therapy, Amivantamab

## Abstract

**背景与目的:**

表皮生长因子受体（epidermal growth factor receptor, *EGFR*）和细胞间质上皮转换因子（cellular-mesenchymal to epithelial transition factor, *c*-*Met*）是非小细胞肺癌（non-small cell lung cancer, NSCLC）中常见的变异基因，都是受体酪氨酸激酶，在下游信号转导方面具有协同作用。针对EGFR和c-Met通路的靶向药物联合应用后，能够阻断PI3K/AKT/mTOR途径和Ras/Raf/Mek途径，限制补偿通路激活，发挥抗肿瘤作用。以Amivantamab为代表的EGFR和c-Met双特异性抗体新型药物能同时阻断两条信号通路，在肺癌临床研究中展现出良好前景。然而目前国内应用较少，相关经验缺乏。本研究将介绍我中心Amivantamab疗效数据和不良反应处理经验，探讨此类药物对我国*EGFR*/*MET*基因异常NSCLC的应用价值。

**方法:**

收集2020年8月-2021年12月于我院接受Amivantamab治疗的晚期NSCLC病例的临床数据，分析其治疗反应率、生存和不良反应。

**结果:**

本研究共纳入15例患者，其中Amivantamab单药治疗6例，Amivantamab联合Lazertinib治疗9例。治疗后7例（46.7%）达部分缓解（partial response, PR），7例（46.7%）达疾病稳定（stable disease, SD），1例（6.7%）达疾病进展（progressive disease, PD），总体客观缓解率（objective response rate, ORR）为46.7%，总体疾病控制率（disease control rate, DCR）为93.3%。7例*EGFR*外显子20插入突变患者中，2例（28.6%）达PR，5例（71.4%）达SD，DCR为100.0%，中位缓解持续时间（duration of response, DOR）未成熟，2例PR者仍持续缓解。5例奥希替尼耐药的患者中，2例（40.0%）达PR，3例（60.0%）达SD，DCR为100.0%，中位DOR未成熟，1例PR者仍持续缓解。中位随访时间为8.7个月（0.9个月-18.6个月），截至最后一次随访，共有6例患者发生疾病进展，3例患者死亡，中位无进展生存期和中位总生存期未成熟。常见的不良事件为皮疹（86.7%），甲沟炎（80.0%）和输液反应（60.0%），多为1级-2级。3级及以上不良反应包括皮疹（33.3%），转氨酶升高（13.3%）、水肿（6.7%）、血栓栓塞（6.7%）、间质性肺炎（6.7%）和血小板减低（6.7%）。

**结论:**

Amivantamab对*EGFR*外显子20插入突变和奥希替尼耐药的中国晚期NSCLC治疗有效，与国外疗效数据相仿。整体安全性可耐受，需重点关注皮疹、甲沟炎、输液反应等不良反应。

## Abstract (en)

**Background and objective:**

Epidermal growth factor receptor (*EGFR*) and cellular-mesenchymal to epithelial transition factor (*c*-*Met*) are widely expressed on cancer cells. There is a synergistic effect of EGFR and HGF/c-Met pathways on proliferation, downstream activation of signal transduction and an additive effect. Studies show that combination of both signaling pathways could potentially be targeted in a synergistic fashion. Amivantamab, a bispecific monoclonal antibody targeting EGFR and c-Met, yielded robust and durable responses in a variety of clinicals trials. However, few researches have reported its efficacy in Chinese non-small cell lung cancer (NSCLC) patients. This study was conducted to evaluate the effectiveness and tolerance of Amivantamab in NSCLC patients with *EGFR*/*MET* gene abnormalities at Peking University Cancer Hospital.

**Methods:**

The study enrolled NSCLC patients who received Amivantamab in our hospital between August 2020 and December 2021, and analyzed the response, survival, and treatment-related adverse events.

**Results:**

Fifteen patients were enrolled in this research, and six of them received Amivantamab treatment and the other nine patients received Amivantamab plus Lazertinib treatment. The rates of partial response (PR), stable disease (SD), and progressive disease (PD) were 46.7% (7/15), 46.7% (7/15) and 6.7% (1/15), respectively. The overall response rate (ORR) and disease control rate (DCR) were 28.6% (2/7) and 100.0% (7/7) in seven patients with *EGFR* exon 20 insertion, respectively. The ORR and DCR were 40.0% (2/5) and 100.0% (5/5) in five post-osimertinib *EGFR*-mutant patients, respectively. After a median follow-up of 8.7 months, the median progression-free survival and overall survival were not reached. The most common treatment-related adverse events were rash (86.7%), paronychia (80.0%), and infusion-related reactions (60.0%), and most of them were graded as 1 to 2. Grade 3 to 4 adverse events included rash (33.3%), alanine aminotransferase elevation (13.3%), gamma-glutamyl transpeptidase elevation (13.3%), peripheral edema (6.7%), thromboembolism (6.7%), interstitial lung disease (6.7%), and thrombocytopenia (6.7%).

**Conclusion:**

Amivantamab was effective in Chinese NSCLC patients with *EGFR* exon 20 insertion and post-Osimertinib *EGFR*-mutant patients, similar to the results of clinical trials conducted in western countries. Amivantamab was well tolerated and emphases should be put on adverse events such as rash, paronychia, and infusion-related reactions.

肺癌是全球发病率和死亡率最高的恶性肿瘤之一，其中非小细胞肺癌（non-small cell lung cancer, NSCLC）患者占总数的85%-90%^[1]^，大部分患者在确诊时已为晚期，预后差，5年生存率低于15%^[2]^。随着基因分析和分子诊断技术研究的深入，多种肺癌驱动基因被发现，表皮生长因子受体（epidermal growth factor receptor, *EGFR*）基因和间质-上皮细胞转化因子（mesenchymal-epithelial transition factor, *MET*）基因是其中重要的两种^[3]^。EGFR是一种跨膜蛋白，属于HER家族的一员，与配体结合后，可形成同源或异源二聚体引起细胞内酪氨酸激酶域活化，随后参与信号通路的细胞内蛋白发生磷酸化而激活，参与肿瘤的生长与发展^[4]^。*MET*基因位于人类7号染色体（7q21-31），由*MET*基因编码的蛋白为c-MET，也称为肝细胞生长因子受体（hepatocyte growth factor receptor, HGFR），肝细胞生长因子（hepatocyte growth factor, HGF）是目前发现的唯一的c-Met配体，HGF能够与c-Met的细胞外结构域结合，促使c-Met发生二聚化、酪氨酸磷酸化，激活众多下游信号通路，从而发挥促进细胞增殖、细胞生长、细胞迁移、侵袭血管及血管生成等效应^[5]^。临床前研究^[6]^发现，EGFR和c-Met都是受体酪氨酸激酶，在下游信号转导方面具有协同作用，EGFR靶向药物和MET靶向药物联合应用后，能够阻断PI3K/AKT/mTOR途径和Ras/Raf/Mek途径，限制补偿通路激活，从而改善总体疗效。

Amivantamab是一种低岩藻糖化、针对EGFR和c-Met的全人源化的基于IgG1的双特异性抗体，通过与肿瘤细胞表面EGFR、c-Met结合阻断EGFR、c-Met信号通路激活，从而阻止肿瘤生长和进展。此外，肿瘤细胞表面存在高水平的EGFR和c-Met，免疫效应细胞可通过抗体依赖性细胞毒性和抗体依赖性细胞吞噬作用机制破坏这些靶细胞。临床前研究^[7-9]^显示Amivantamab对具有原发性*EGFR*激活突变、T790M耐药突变、过表达野生型EGFR和c-Met通路激活的肿瘤具有抑制活性。临床研究证实该药不仅对携带*EGFR*敏感突变、奥希替尼耐药患者具有良好疗效，而且对于*EGFR*外显子20插入突变NSCLC也较为敏感。目前国内有类似的EGFR/c-Met双抗正在Ⅰ期-Ⅱ期临床研究阶段，预计不久有望获批国内临床。可见，双抗药物未来可能成为*EGFR*/*MET*基因异常NSCLC治疗的重要手段。

但是目前此类药物在国内使用经验较少，本研究总结了我中心使用Amivantamab治疗NSCLC的疗效和不良反应数据，希望与国内同行分享经验，以期提高相关的临床治疗和安全管理水平。

## 1 对象与方法

### 1.1 研究对象

收集2020年8月-2021年12月于我院接受Amivantamab治疗的晚期NSCLC病例。纳入标准：①年龄≥18岁；②东部肿瘤协作组体能状态评分为0分或1分；③经组织学或细胞学证实的局部晚期或转移性NSCLC；④具有*MET*基因或*EGFR*基因变异；⑤基线期至少存在一处可测量的靶病灶；⑥有充分的器官功能。排除标准：①年龄 < 18岁；②合并其他恶性肿瘤；③妊娠期；④未得到控制的其他疾病。患者来自3项临床研究：NCT02609776、NCT04077463和NCT04487080。治疗剂量及剂量调整方法均按照入组的剂量组和试验要求进行。

### 1.2 基因检测方法

*EGFR*变异检测方法包括基于循环肿瘤DNA（circulating tumor DNA, ctDNA）或肿瘤组织的二代测序，基于肿瘤组织的聚合酶链式反应（polymerase chain reaction, PCR）。*MET*外显子14跳跃突变的检测方法包括基于ctDNA或肿瘤组织的二代测序或反转录PCR。*MET*扩增采用荧光原位杂交和基于肿瘤组织的二代测序进行检测。

### 1.3 统计学方法

采用SPSS 22.0软件进行统计学分析。依据实体瘤疗效评价标准1.1标准进行肿瘤治疗疗效的评估。通过不良反应术语标准5.0评价毒副反应。疗效评价指标包括完全缓解（complete response, CR）、部分缓解（partial response, PR）、疾病稳定（stable disease, SD）和疾病进展（progressive disease, PD）。客观缓解率（objective response rate, ORR）=（CR+PR）/总例数×100%，疾病控制率（disease control rate, DCR）=（CR+PR+SD）/总例数×100%。两组间ORR比较采用卡方检验或*Fisher*法检验。无进展生存期（progression-free survival, PFS）指患者从入组开始，到观察到疾病进展或发生因任何原因死亡或末次随访的时间。总生存期（overall survival, OS）为从入组开始至因任何原因死亡或末次随访的时间。采用*Kaplan-Meier*法分析患者的PFS和OS，*Log-rank*检验进行两组生存比较。*P* < 0.05为差异有统计学意义。

## 2 结果

### 2.1 临床病理特征

如表 1所示，本研究共纳入15例患者，其中Amivantamab单药治疗6例，Amivantamab联合Lazertinib治疗9例。治疗期间定期复查直至疾病进展或无法耐受不良反应。入组患者中，男性6例（40.0%, 6/15），女性9例（60.0%, 9/15）。≤60岁者占46.7%（7/15），61岁-69岁者占40.0%（6/15），≥70岁者占13.3%（2/15）。不吸烟者占73.3%（11/15）。按照第八版肿瘤原发灶-淋巴结-转移（tumor-node-metastasis, TNM）分期，所有患者入组时均已出现局部或远处转移，其中有脑转移者占46.7%（7/15）。Amivantamab治疗为一线治疗者占33.3%（5/15）。既往接受含铂类化疗失败者占60.0%（9/15），既往接受免疫治疗失败的占26.7%（4/15），既往接受酪氨酸激酶抑制剂（tyrosine kinase inhibitor, TKI）靶向治疗的占40.0%（6/15）。

**表 1:** 15例接受Amivantamab治疗NSCLC患者的临床特点，治疗和转归（*n*=15） Clinicopathologic characteristics, treatment and prognosis of non-small cell lung cancer patients with Amivantamab treatment (*n*=15)

No.	Gender	Age (yr)	Smoking	Histology	Stage	*EGFR*/*MET* gene	Therapy lines of Amivantamab	Treatment	Platinum-relapsed	ICI- relapsed	TKI-relapsed	With brain metastases	Efficacy of Amivantamab	PFS	OS
1	Female	68	No	ASC	IV	*EGFR* 20ins	3	Amivantamab	Yes	Yes	No	Yes	PR	18.6^a^	18.6^b^
2	Female	61	No	ADC	IV	*EGFR* 20ins	1	Amivantamab	No	No	No	No	SD	17.5^a^	17.5^b^
3	Female	68	No	ADC	IV	*EGFR* L858R, T790M, cis-C797S	5	Amivantamab	Yes	No	Gefitinib; Osimertinib	Yes	PR	14.4	14.4
4	Female	62	No	ADC	IV	*EGFR* L858R, T790M, cis-C797S	5	Amivantamab	Yes	No	Gefitinib; Osimertinib	No	SD	3.5	8.7
5	Male	72	No	ADC	IV	*EGFR* 20ins	2	Amivantamab	Yes	No	No	No	SD	5.1	15.7^b^
6	Male	62	No	ADC	IV	*MET* ex14	5	Amivantamab	Yes	Yes	Savolitinib; Glumetinib	Yes	PD	0.8	0.9
7	Female	53	No	ADC	IV	*EGFR* 20ins	3	Amivantamab+ Lazertinib	Yes	Yes	No	No	SD	7.7^a^	7.7^b^
8	Male	52	Yes	ADC	IV	*EGFR* 20ins	2	Amivantamab+ Lazertinib	Yes	No	No	No	SD	4.3	7.0^b^
9	Male	57	Yes	ADC	IV	*EGFR* L861Q	1	Amivantamab+ Lazertinib	No	No	No	Yes	PR	6.5^a^	6.5^b^
10	Male	65	Yes	ADC	IV	*EGFR* 20ins	1	Amivantamab+ Lazertinib	No	No	No	No	PR	4.0^a^	4.0^b^
11	Female	52	No	ADC	IV	*EGFR* 19del, *EGFR* T790M, *MET* amplification	4	Amivantamab+ Lazertinib	Yes	No	Icotinib；Osimertinib	Yes	PR	11.1^a^	11.1^b^
12	Female	55	No	ADC	IV	*EGFR* 19del, *EGFR* T790M	5	Amivantamab+ Lazertinib	Yes	Yes	Gefitinib; Osimertinib	Yes	SD	9.1^a^	9.1^b^
13	Female	70	No	ADC	IV	*EGFR* 20ins	2	Amivantamab+ Lazertinib	No	No	Osimertinib	No	SD	7.9^a^	7.9^b^
14	Male	54	Yes	ADC	IIIC	*EGFR* L858R	1	Amivantamab+ Lazertinib	No	No	No	No	PR	5.4	9.2^b^
15	Female	35	No	ADC	IV	*EGFR* 19del, *EGFR* amplification, *MET* amplification	1	Amivantamab+ Lazertinib	No	No	No	Yes	PR	5.6^a^	5.6^b^
^a^: These patients were still receiving Amivantamab treatment, and PFS was defined as the period from the date of enrollment to the date of last time follow up; ^b^: These patients were still alive, and OS was defined as the period from the date of enrollment to the date of last time follow up. NSCLC: non-small cell lung cancer; ASC: adenosquamous carcinoma; ADC: adenocarcinoma; EGFR: epidermal growth factor receptor; MET: mesenchymal epithelial transition; ICI: immune checkpoint inhibitor; TKI: tyrosine kinase inhibitor; PFS: progression-free survival; OS: overall survival.															

### 2.2 基因突变情况

15例患者中，*EGFR*外显子20插入突变7例（7/15），*MET*外显子14跳跃突变者1例（1/15），*EGFR* L858R阳性者1例（1/15），*EGFR* L858R阳性且T790M阳性且C797S阳性者2例（2/15），*EGFR* L861Q阳性者1例（1/15），*EGFR*外显子19缺失突变且*MET*扩增且*EGFR*扩增者1例（1/15），*EGFR*外显子19缺失突变且T790M阳性者1例（1/15），*EGFR*外显子19缺失突变且T790M阳性且*MET*扩增者1例（1/15）。

### 2.3 疗效分析

如表 1和图 1所示，15例患者中近期疗效0例达到CR，7例（46.7%）达到PR，7例（46.7%）达到SD，1例（6.7%）达到PD，总体ORR为46.7%，总体DCR为93.3%，最佳疗效评估时间为0.6个月-11.0个月（图 1）。一线接受Amivantamab单药或联合Lazertinib治疗者5例，4例（80.0%）达到PR，1例（20.0%）达到SD，ORR为80.0%。二线到五线接受治疗者10例，3例（30.0%）达到PR，6例（60.0%）达到SD，1例（10.0%）达到PD，ORR为30.0%。一线应用Amivantamab的ORR更高（80.0% *vs* 30.0%），但是这种差异没有统计学意义（*P*=0.200）。7例*EGFR*外显子20插入突变患者中，2例达PR（28.6%），5例达SD（71.4%），DCR为100.0%，中位缓解持续时间（duration of response, DOR）未成熟，2例PR者仍持续缓解。5例奥希替尼耐药的患者中，2例达PR（40.0%），3例达SD（60.0%），DCR为100.0%，中位DOR未成熟，1例PR者仍持续缓解。末次随访时间为2022年4月30日，中位随访时间为8.7个月（0.9个月- 18.6个月），截至最后一次随访，共有6例患者发生PD，3例患者死亡，中位PFS和中位OS尚未达到。近期疗效为PR、SD、PD者的PFS（中位PFS：14.4个月*vs*未达到*vs* 0.8个月，*P*=0.001）和OS（中位OS：14.4个月*vs*未达到*vs* 0.9个月，*P* < 0.001）可见统计学差异。

**图 1:**
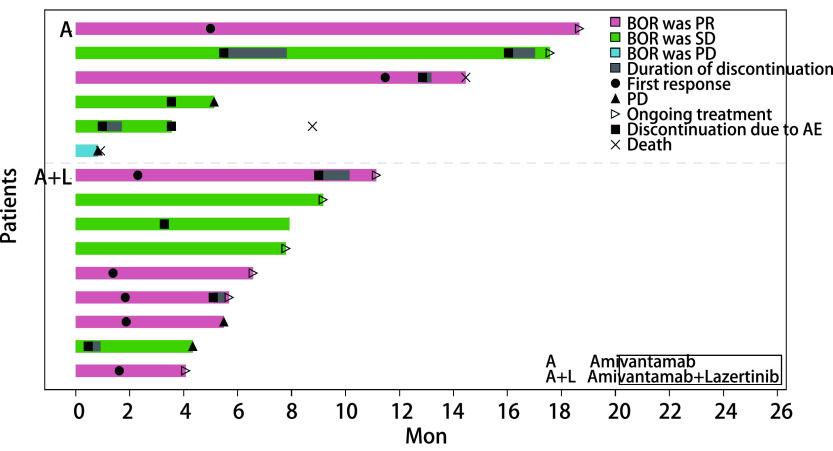
所有入组患者的电泳图（*n*=15） Swimmer plot of all enrolled patients (*n*=15)

BOR: best of response; PR: partial response; SD: stable disease; PD: progressive disease; AE: adverse event

### 2.4 不良反应

表 2列出Amivantamab单药或联合Lazertinib治疗后，考虑与治疗药物相关的发生例数≥2例或者可见≥3级的不良反应。常见的不良事件有皮疹（86.7%），甲沟炎（80.0%）和输液反应（60.0%）。治疗前给予地塞米松、苯海拉明、对乙酰氨基酚、雷尼替丁等进行预处理，输液相关反应一般出现在第一次输注，表现为咳嗽、气短、胸闷、恶心、面部潮红等，对症治疗缓解后，不影响后续治疗。若治疗过程中，患者停药超过1个月，则需要重新预处理。皮疹（图 2）多见于颜面部及躯干，部分可波及全身，伴瘙痒，重者可见局部破溃及皲裂，给予曲安奈德益康唑软膏、莫匹罗星软膏、氢化可的松软膏、丙酸氟替卡松乳膏、碘伏等对症治疗后好转。甲沟炎（图 2）表现为甲沟肿胀或红斑，甲周皮肤受损，部分甲板分离，保持皮肤干燥，使用滋润霜，外用抗生素或抗真菌药物，必要时可外用糖皮质激素药物等治疗。3级及以上不良反应包括皮疹（33.3%）、谷丙转氨酶（alaninetransaminase, ALT）升高（13.3%），谷草转氨酶（aspartate aminotransferase, AST）升高（13.3%）、水肿（6.7%）、血栓栓塞（6.7%）、间质性肺炎（6.7%）、血小板减低（6.7%）。治疗过程中有10例患者停药，部分原因与试验药物无关，如右肾囊肿出血、感染性肺炎、痔疮出血，与治疗药物无关的不良反应好转后原量恢复用药。图 1中标出了与试验药物相关的不良反应导致的停药。1例因3级皮疹停药一次，之后减量恢复用药，因3级甲沟炎合并2级口腔溃疡停药一次，之后减量恢复用药；另1例因肺栓塞停药一次，之后原量恢复用药，因间质性肺炎停药一次，之后停药出组。另外6例因药物相关不良反应各停药一次，3例因3级皮疹停药一次，其中1例停药后疾病进展出组，2例减量恢复用药；1例因3级皮疹合并3级水肿停药一次，之后未恢复用药；1例因2级皮疹长期不恢复停药一次，之后减量恢复用药；1例因3级血小板减少停药一次，之后原量恢复用药。无治疗相关死亡。

**表 2:** Amivantamab或Lazertinib+Amivantamab治疗相关不良事件[*n*=15, *n* (%)] Treatment-related adverse events of Amivantamab with or without Lazertinib [*n*=15, *n* (%)]

Adverse events	All grades	≥Grade 3
Rash	13 (86.7)	5 (33.3)
Paronychia	12 (80.0)	0
Infusion-related reaction	9 (60.0)	0
Hypoalbuminemia	8 (53.3)	0
Increased alanine aminotransferase	7 (46.6)	2 (13.3)
Increased gamma-glutamyl transpeptidase	6 (40.0)	2 (13.3)
Peripheral edema	6 (40.0)	1 (6.7)
Stomatitis	6 (40.0)	0
Acid regurgitation	5 (33.3)	0
Leukopenia	5 (33.3)	0
Abdominal distension	4 (26.7)	0
Anemia	3 (20.0)	0
Thromboembolism	3 (20.0)	1 (6.7)
Conjunctivitis	3 (20.0)	0
Constipation	3 (20.0)	0
Gingival bleeding	3 (20.0)	0
Decreased appetite	3 (20.0)	0
Fatigue	3 (20.0)	0
Increased aspartate aminotransferase	3 (20.0)	0
Diarrhea	2 (13.3)	0
Numbness of the limbs	2 (13.3)	0
Hypocalcemia	2 (13.3)	0
Pneumonia	2 (13.3)	0
Hyperbilirubinemia	2 (13.3)	0
Interstitial lung disease	1 (6.7)	1 (6.7)
Thrombocytopenia	1 (6.7)	1 (6.7)

**图 2:**
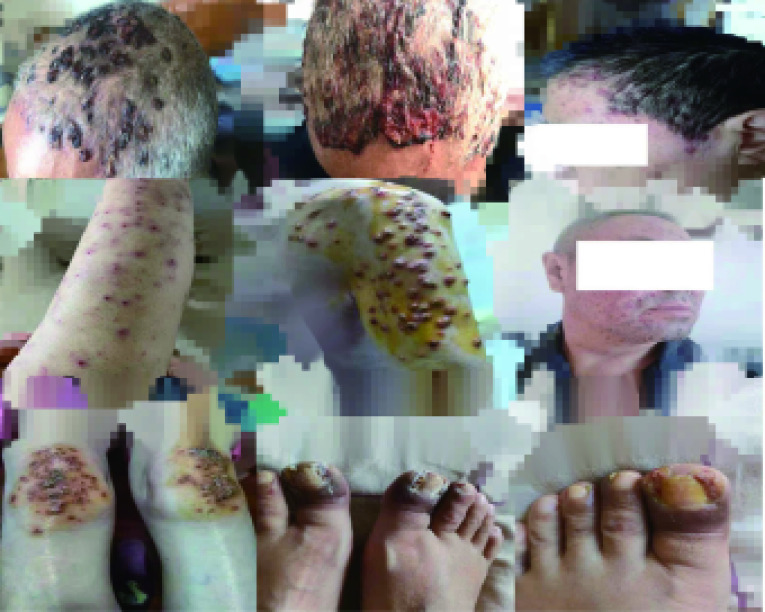
治疗相关不良反应（皮疹和甲沟炎） Treatment-related AEs (rash and paronychia)

## 3 讨论

本研究是目前首次报道此类药物在中国NSCLC患者中的治疗经验。本研究数据显示，Amivantamab单药或联合Lazertinib在初治患者的中的ORR可达80%（4/5），DCR可达100.0%（5/5），在EGFR-TKI耐药后的患者中的ORR可达30%（3/10），中位PFS超过6.5个月。初步显示了该药物在中国患者中的疗效。

Amivantamab治疗*EGFR*外显子20插入突变的NSCLC患者疗效突出，已经获批国外临床。CHRYSALIS研究^[9]^是一项开放标签、多中心的I期临床研究，评估了Amivantamab治疗晚期NSCLCL患者的安全性、药代动力学和疗效，该研究设计多个队列，其中队列D纳入经含铂化疗治疗失败后的*EGFR*外显子20插入突变NSCLC患者，评估后线使用Amivantamab的疗效及安全性。该队列研究结果显示在疗效评估人群的81例患者中，ORR为40%，中位DOR为11.1个月，临床获益率为74%，中位PFS为8.3个月，中位OS为22.8个月。此外，Amivantamab也被证实对于不同插入区域的外显子20插入突变亚型均有效。基于此研究结果，2021年美国食品药品监督管理局加速批准Amivantamab上市，用于治疗铂类化疗后进展的*EGFR*外显子20插入突变的转移性NSCLC患者。本研究中入组7例*EGFR*外显子20插入突变患者，2例达PR，5例达SD，ORR为40%，目前5例患者仍在治疗中，中位治疗时间已超过6.5个月。疗效接近国外已发表数据，但仍然需要更长时间随访积累更多的生存数据。

对于奥希替尼耐药后的NSCLC患者，目前临床治疗措施有限。2021年欧洲肿瘤内科学会（European Society for Medical Oncology, ESMO）年会报道了CHRYSALIS研究结果^[7]^，该研究是最早将Amivantamab用于奥希替尼耐药后的探索试验，采用了单药或与三代EGFR靶向药Lazertinib联合方案。结果显示，单药的ORR为19%，DCR为48%，中位DOR为5.9个月；联合方案的ORR为36%，DCR为61%，中位DOR为9.6个月，证实了Amivantamab在奥希替尼耐药后的临床挽救疗效。同期ESMO公布的另外一项研究（CHRYSALIS-2研究）^[8]^进一步探索了Amivantamab联合Lazertinib治疗既往奥希替尼和化疗耐药的*EGFR*突变的晚期肺癌患者，并按三线/四线用药患者和既往重度治疗患者进行分组评估。结果显示，在既往奥希替尼和化疗失败后、按照三线/四线用药入组的患者，ORR为41%，DCR为69%。在既往重度治疗的患者中，ORR为21%，DCR为51%。无论在标准三线/四线还是更重度治疗的患者，Amivantamab联合Lazertinib都能创造出可观的疗效。在本研究中，入组6例奥希替尼/谷美替尼耐药后的患者，接受Amivantamab单药或者联合Lazertinib治疗，其中2例PR，3例SD，1例PD，3例患者仍在治疗中，中位治疗时间超过6个月。初步数据与国外结果相接近，提示该方案未来有望成为我国奥希替尼耐药患者的后续治疗选择，但仍需进一步随访。

*MET*异常导致肿瘤增殖、迁移、血管生成等效应^[5, 10, 11]^。研究^[12, 13]^显示，60%-80%的NSCLC患者存在c-MET的过度激活。NSCLC中*MET*外显子14跳跃突变的总体发生率为3%-4%^[14]^，奥希替尼耐药后的NSCLC中有大约20%会出现*MET*扩增。CHRYSALIS研究^[15]^显示，Amivantamab治疗*MET*外显子14跳跃突变NSCLC的ORR达64%，中位治疗持续时间6.5个月。本研究中有3例患者出现*MET*异常，其中2例*MET*扩增，1例*MET*外显子14跳跃突变，结果显示2例PR，1例PD，样本量较小，故需进一步积累数据。

*EGFR*/*MET*变异的NSCLC患者常常出现脑转移。Amivantamab作为一个靶向EGFR的大分子抗体，其对血脑屏障的穿透性待进一步研究。CHRYSALIS系列研究^[16]^中纳入114例*EGFR*外显子20插入突变的NSCLC患者，其中38例在基线合并脑转移，随访12.5个月后，基线有脑转移的患者治疗过程中出现颅内病灶进展的几率明显高于基线无脑转移的患者（32% *vs* 6.6%）。本研究中，单药Amivantamab治疗组中有3例基线合并脑转移，颅内转移灶1例SD，1例non-CR/non-PD，1例PD，而Amivantamab联合Lazertinib治疗组颅内病灶的CR率可达到50.0%（2/4），DCR可达100.0%（4/4）。与之相比，小分子MET抑制剂在*MET*外显子14跳跃突变的肺癌脑转移患者中显示出明显疗效，颅内病灶控制率可达92.3%^[17, 18]^。因此，对于有脑转移的患者，小分子MET抑制剂或者Amivantamab联合Lazertinib治疗可能是更好的选择。

在安全性方面，本研究中常见的不良反应为皮疹（86.7%）、甲沟炎（80.0%）和输液反应（60.0%）。皮疹和甲沟炎临床表现和处理同EGFR-TKI导致的皮肤不良反应。输液反应主要出现在第1周期第1天首次给药时，通常发生于输注开始后最初90 min内，多为1级-2级，与Amivantamab免疫原性相关，积极预处理和减慢输液速度可减轻。Amivantamab的治疗相关不良反应谱与小分子MET抑制剂有明显不同，后者消化系统症状和肝功能异常更多见^[17, 18]^，提示MET抗体药物与MET-TKI的不良反应处理应当有所区别。

本研究存在一些局限性：①本研究中患者的基因异常类型多样，样本量小，无法针对各种异常类型进行分类统计分析，因此研究结论需要更进一步的验证；②Amivantamab治疗后仍不可避免的产生耐药，本研究未对耐药机制进一步的探索。文献报道耐药机制复杂多样，包括原有*MET*基因的改变，新突变的出现，其他基因的激活，下游信号通路的改变等等。这是我们下一步探索的方向。

本研究创新之处在于首次报道了中国NSCLC患者应用EGFR和c-Met双特异性抗体Amivantamab的临床经验，初步证实对*EGFR*外显子20插入突变或奥希替尼耐药的中国NSCLC患者有效。但需关注皮疹、甲沟炎、输液反应等不良反应。相信随着研究的进一步开展和深入，积累更大样本的经验，此类药物有望改变*EGFR*突变患者的治疗现状，为*MET*基因或*EGFR*基因异常的NSCLC患者带来更多的治疗机会。
